# Effects of Prophylactic Administration of Granulocyte Colony-Stimulating Factor on Peripheral Leukocyte and Neutrophil Counts Levels After Chemotherapy in Patients With Early-Stage Breast Cancer: A Retrospective Cohort Study

**DOI:** 10.3389/fonc.2022.777602

**Published:** 2022-04-25

**Authors:** Wei Tian, Yali Wang, Yunxiang Zhou, Yihan Yao, Yongchuan Deng

**Affiliations:** ^1^ Department of Breast Surgery, The Second Affiliated Hospital, School of Medicine, Zhejiang University, Hangzhou, China; ^2^ Institute of Immunology, School of Medicine, Zhejiang University, Hangzhou, China

**Keywords:** early-stage breast cancer, white blood cell counts, absolute neutrophil counts, chemotherapy-induced leukopenia, chemotherapy-induced neutropenia

## Abstract

**Background:**

Both chemotherapy-induced neutropenia (CIN) and febrile neutropenia (FN) frequently occur and can lead to dose-limiting toxicity and even fatal chemotherapy side effects. The prophylactic use of recombinant human granulocyte colony-stimulating factor (rhG-CSF), including pegylated rhG-CSF (PEG-rhG-CSF), significantly reduces the risks of CIN and FN during chemotherapy in early-stage breast cancer (ESBC) patients. However, whether the prophylactic use of granulocyte colony-stimulating factor (G-CSF), especially PEG-rhG-CSF, can influence white blood cell (WBC) counts and absolute neutrophil counts (ANCs) after finishing the chemotherapy remains unknown. Therefore, exploring the development and recovery tendency of WBC counts and ANCs during and after chemotherapy is crucial.

**Objective:**

We aimed to investigate the variation tendency and recovery of WBC counts and ANCs during and after chemotherapy and evaluate the independent factors influencing leukopenia and neutropenia lasting longer after chemotherapy. We also aimed to provide individualized prophylactically leukocyte elevation therapy for breast cancer patients.

**Methods:**

This single-center retrospective cohort study evaluated 515 ESBC patients who received rhG-CSF or PEG-G-CSF for prophylaxis after adjuvant or neoadjuvant chemotherapy. Blood test reports were analyzed during chemotherapy, and on a 12-month follow-up period after finishing the chemotherapy. The WBC counts and ANCs were measured to assess their variation tendency characteristics and to identify independent factors that influenced the occurrence of leukopenia and neutropenia lasting longer than 12 months after chemotherapy.

**Results:**

Prophylaxis with rhG-CSF or PEG-rhG-CSF kept the mean values of WBC counts and ANCs within the normal range during chemotherapy, but a significant difference in WBC levels was detected before the end of the last chemotherapy compared to the prechemotherapy period (baseline) (*p* < 0.001). During the 12-month follow-up after the end of the last chemotherapy, WBC counts and ANCs gradually recovered, but the group that used only PEG-rhG-CSF (long-acting group, *p*_WBC_ = 0.012) or rhG-CSF (short-acting group, *p*_WBC_ = 0.0005) had better leukocyte elevation effects than the mixed treatment group (PEG-rhG-CSF mixed rhG-CSF). Besides, the short-acting group had a better neutrophil elevation effect than the longer-acting (*p*_ANC_ = 0.019) and mixed (*p*_ANC_ = 0.002) groups. Leukopenia was still present in 92 (17.9%) patients and neutropenia in 63 (12.2%) 12 months after the end of the last chemotherapy. The duration of leukopenia over 12 months was closely associated with the baseline WBC level (*p* < 0.001), G-CSF types (*p* = 0.027), and surgical method (*p* = 0.041). Moreover, the duration of neutropenia over 12 months was closely related to the baseline ANC (*p* < 0.001), G-CSF types (*p* = 0.043), and molecular typing (*p* = 0.025).

**Conclusion:**

The prophylactic application of G-CSF effectively stabilized the WBC counts and ANCs during chemotherapy in ESBC patients. Nevertheless, the recovery of WBC counts and ANCs after chemotherapy varied between different G-CSF treatment groups. The risk of leukopenia and neutropenia persisting for more than 12 months after chemotherapy was associated with G-CSF types, the baseline level of WBC count/ANCs, surgical method, and molecular typing.

## 1 Introduction

Breast cancer ranks first among female malignancies worldwide (1). Neoadjuvant and adjuvant chemotherapies are important treatments for early-stage breast cancer (ESBC) patients (2). Sufficient dose and course of chemotherapy can effectively reduce the risk of breast cancer recurrence and significantly improve overall survival (OS) and disease-free survival (DFS) (3). Nevertheless, chemotherapies can cause different toxic and side effects, including chemotherapy-induced neutropenia (CIN) and febrile neutropenia (FN), which limits the continuous relative dose intensity (RDI) of the treatment (4, 5).

Leukopenia and neutropenia are common chemotherapy side effects caused by the reduction of white blood cell (WBC) counts and absolute neutrophil counts (ANCs) during myelosuppressive chemotherapeutic treatments (6). Moreover, CIN and FN might lead to dose schedule alterations, treatment delays, elevated risk of infection, increased medical costs, prolonged treatment time, and even life-threatening complications (7, 8). Therefore, it is crucial to supervise the variation tendency of WBC counts and ANCs during and post-chemotherapy, as well as explore the independent factors influencing CIN.

The occurrence of CIN and FN during chemotherapy is closely related to chemotherapy regimen and intensity (9). However, these treatment-related factors do not have effective preventive approaches. Additionally, patient-related risk factors should also be considered.

Furthermore, the granulocyte colony-stimulating factor (G-CSF)—the principal cytokine responsible for the regulation of neutrophil production—boosts neutrophil counts by controlling the maturation, proliferation, and differentiation of hematopoietic stem cells and modulating bone marrow (BM) neutrophils into the circulation (10–12). Previous trials have demonstrated that prophylaxis with G-CSF after chemotherapy in breast cancer patients can effectively decrease neutropenia incidence and complications and improve the tolerance to the treatment (13). Recombinant human G-CSF (rhG-CSF) exhibits a short half-life due to its primary clearance through the kidney. To prolong its half-life, PEG was introduced into rhG-CSF (PEG-rhG-CSF), changing the clearance approach and reducing systemic clearance rates (14, 15). PEG-rhG-CSF is as safe and effective as rhG-CSF to reduce CIN in breast cancer (16). It can also reduce the number of injections and improve life quality and therapeutic compliance. On the other hand, rhG-CSF has lower prices and quicker effects (17).

Many studies have indicated that age, baseline, cancer type, cancer stage, and molecular typing can influence the occurrence of CIN and FN during chemotherapy (18, 19). Meanwhile, in our clinical practice, ESBC patients have been routinely treated with G-CSF for prophylaxis after the each cycle of chemotherapy. However, in some patients, leukopenia and neutropenia lasted for more than 12 months after the end of the last chemotherapy. The specific reasons for this long duration of chemotherapy-induced leukopenia (CIL) and CIN remain unknown, and current trials are missing and few. Therefore, it is necessary to study the risk factors for the long duration of leukopenia and neutropenia after the end of the last chemotherapy to guide G-CSF use. The present retrospective cohort study was conducted to analyze the variation tendency of WBC counts and ANCs during chemotherapy and in the 12-month follow-up period after the end of the last chemotherapy. We also compared the effects of the prophylactic use of different G-CSF types on the recovery of WBC counts and ANCs and explored independent factors influencing the long duration of leukopenia and neutropenia after the last chemotherapy to guide the use of G-CSF during chemotherapy.

## 2 Material and Methods

### 2.1 Study Participants and Design

**Figure 1:**
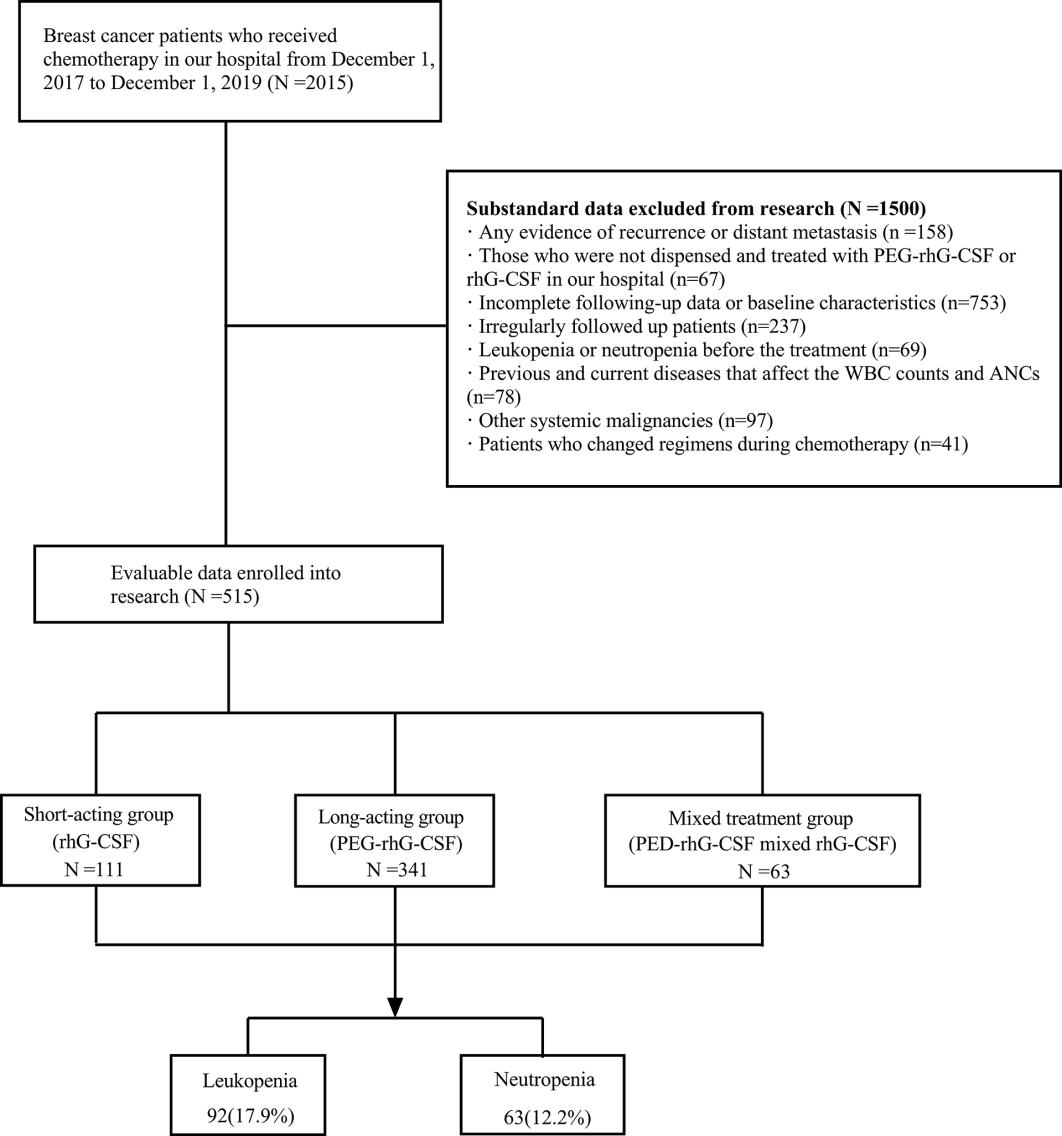
Diagram of the participants included and excluded in analyses. This study collected 2015 breast cancer patients who received chemotherapy in our hospital from November 1, 2017, to November 1, 2019. Among them, 1,500 did not meet the criteria, and 515 were finally enrolled. PEG-rhG-CSF, pegylated recombinant human granulocyte colony-stimulating factor; rhG-CSF, recombinant human granulocyte colony-stimulating factor; N, number.

A retrospective analysis was performed based on our prospectively collected 2015 patients with breast cancer who received adjuvant or neoadjuvant chemotherapy at the Second Affiliated Hospital of Zhejiang University School of medicine from November 1, 2017, to November 1, 2019. A total of 1,500 patients were excluded because of the following reasons: 1) were not at early-stage, namely, had any evidence of recurrence or distant metastasis; 2) were not dispensed and treated with PEG-rhG-CSF or rhG-CSF in our hospital; 3) had a follow-up time lower than 12 months after the last chemotherapy; 4) had incomplete baseline characteristics data; 5) were not regularly followed up as required; 6) suffered leukopenia or neutropenia before the treatment; 7) had a diagnosis of diseases that affect the WBC counts and ANCs, such as blood, immune, and infectious diseases; 8) underwent other systemic malignancies; and 9) changed their chemotherapy regimens during treatment (**Figure 1**).

All enrolled patients received G-CSF for prophylaxis during each cycle of chemotherapy. The G-CSF types and application methods were 1) 100-μg subcutaneous injection of rhG-CSF (Qilu Pharmaceutical, Jinan, China) once a day for 5–7 days and 2) 3- or 6-mg subcutaneous injections of PEG-rhG-CSF (GSPC Pharmaceutical Group Limited or Qilu Pharmaceutical) 48 h after each cycle of chemotherapy.

The demographic data of the 515 patients were prospectively collected, including age, body mass index (BMI), G-CSF types, baseline levels of WBC counts and ANCs before treatment, surgical method, pathological type, molecular typing, lymph node metastasis, chemotherapy regimen, and Herceptin use, and WBC counts and ANCs during chemotherapy and the 12-month follow-up period after the end of the last chemotherapy (3, 6, 9, and 12 months). Patients were divided into four groups according to different chemotherapy regimens and stratified according to G-CSF types. Blood routine results were collected and analyzed from the electronic medical record system (EMRS) database of the Second Affiliated Hospital of Zhejiang University School of Medicine.

### 2.2 Statistical Analyses

Statistical analyses were performed using SPSS 26.0. Two-tailed significance values were used, and a *p* < 0.05 was considered statistically different. The least significant difference (LSD) *t*-test was used for multiple comparisons. Fisher’s exact tests were used to compare the differences of categorical variables between groups. For continuous variables, independent sample two-tailed t-tests or Mann–Whitney U tests were used to compare the differences in median values between groups. Multi-pair sample non-parametric (Friedman) tests were used to describe the variation trend in WBC counts and ANCs during chemotherapy and the 12-month follow-up period after the end of the last chemotherapy. Repeated-measures ANOVA was used to compare inter- and intra-group differences between G-CSF treatment groups during the 12-month follow-up period after the end of the last chemotherapy. To analyze the independent factors influencing leukopenia and neutropenia 12 months after the end of the last chemotherapy, the variables that were potentially correlated with the development of leukopenia/neutropenia and had a univariate *p* < 0.1 were used in a multivariable logistic regression model.

## 3 Results

### 3.1 Characteristics of the Study Population

A total of 515 patients were enrolled in the present study. Their basic clinical characteristics are shown in **Table 1**. After G-CSF prophylactic medication during chemotherapy cycles, some patients developed leukopenia and/or neutropenia until 12 months after the end of the last chemotherapy. The table displays the comparison of univariate characteristics of WBC counts and ANCs influencing factors in ESBC after chemotherapy, as well as the baseline clinical characteristics related to the risk of leukopenia/neutropenia at the 12th month after the end of the last chemotherapy.

**Table 1:** Univariate characteristics of patients in 12 months after the end of the last chemotherapy.

Variables	Baseline characteristics (N, %)	Leukopenia in 12th month (N, %)	Univariate analysis of leukopenia		Neutropenia in 12th month (N, %)	Univariate analysis of neutropenia	
			OR (95% CI)	*p*-Value		OR (95% CI)	*p*-Value
**BMI**	515	92	0.94 (0.86~1.02)	0.118	63	0.98 (0.89~1.07)	0.616
**WBC/ANC baseline level**	515	92	0.49 (0.40-0.60)	<0.0001^*^	63	0.46 (0.34~0.60)	<0.0001^*^
**Age (year)**							
**≤35**	36 (7%)	4 (4.3%)	0.55 (0.16~1.86)	0.337	3 (4.8%)	0.75 (0.18~3.11)	0.695
**35-60**	414 (80.4%)	76 (82.7%)	0.99 (0.51~1.95)	0.984	53 (84.1%)	1.22 (0.53~2.81)	0.646
**>60^#^**	65 (12.6%)	12 (13.0%)			7 (11.1%)		
**G-CSF**							
**Short-acting G-CSF**	111 (21.6%)	14 (15.2%)	0.33 (0.15~0.73)	0.006^*^	11 (17.5%)	0.42 (0.17~1.01)	0.053^*^
**Long-acting G-CSF**	341 (66.2%)	59 (64.1%)	0.49 (0.26~0.89)	0.019^*^	39 (61.9%)	0.50 (0.25~0.99)	0.049^*^
**Mixed group^#^**	63 (12.2%)	19 (20.7%)			13 (20.6%)		
**Surgical method**							
**BCS+SLNB**	130 (25.2%)	22 (23.9%)	0.71 (0.40~1.26)	0.237	18 (28.6%)	1.28 (0.65~2.51)	0.475
**BCS+ALND**	49 (9.5%)	10 (10.9%)	0.89 (0.41~1.93)	0.771	6 (9.5%)	1.11 (0.42~2.92)	0.833
**Mastectomy+SLNB**	148 (28.7%)	18 (19.6%)	0.48 (0.26~0.88)	0.017^*^	18 (28.6%)	1.10 (0.56~2.15)	0.778
**Mastectomy+ALND^#^**	188 (36.5%)	42 (45.6%)			21 (33.3%)		
**Pathological type**							
**Non-specific type of invasive carcinoma**	448 (87%)	80 (87.0%)	1.00 (0.51~1.95)	0.992	57 (90.5%)	1.48 (0.61~3.59)	0.383
**Specific type of invasive carcinoma^#^**	67 (13%)	12 (13.0%)			6 (9.5%)		
**Molecular typing**							
**HR+(Her-2−)**	225 (43.7%)	35 (38.0%)	1.09 (0.54~2.22)	0.812	28 (44.5%)	1.82 (0.72~4.58)	0.200
**HR+(Her-2+)**	109 (21.2%)	25 (27.2%)	1.76 (0.83~3.76)	0.143	21 (33.3%)	3.06 (1.18~7.98)	0.022^*^
**HR-(Her-2+)**	98 (19%)	20 (21.8%)	1.52 (0.69~3.33)	0.298	8 (12.7%)	1.14 (0.38~3.43)	0.815
**TNBC^#^**	83 (16.1%)	12			6 (9.5%)		
**Lymphatic metastasis**							
**N0**	323 (62.7%)	53 (57.6%)	0.28 (0.08~0.90)	0.033^*^	42 (66.7%)	0.75 (0.16~3.53)	0.713
**N1 (1–3)**	131 (25.4)	23 (25.0%)	0.30 (0.09~1.02)	0.054^*^	11 (17.5%)	0.46 (0.09~2.36)	0.351
**N2 (4–9)**	49 (9.5%)	11 (12.0%)	0.41 (0.11~1.53)	0.183	8 (12.7%)	0.98 (0.18~5.32)	0.977
**N3 (≥10)**	12 (2.3%)	5 (5.4%)			2 (3.1%)		
**Chemotherapy regimens**							
**4EC-4T**	206 (40%)	46 (50.0%)	1.51 (0.81~2.83)	0.198	23 (36.5%)	0.92 (0.44-1.94)	0.830
**4TC**	170 (33%)	23 (25.0%)	0.82 (0.41~1.64)	0.577	22 (34.9%)	1.09 (0.51~2.31)	0.822
**6TC**	39 (7.6%)	7 (7.6%)	1.15 (0.43-3.05)	0.781	6 (9.5%)	1.33 (0.46~3.84)	0.594
**6TCbH(P)^#^**	100 (19.4%)	16 (17.4%)			12 (19.1%)		
**Anti-HER-2 targeted therapy**							
**Yes**	204 (39.6%)	44 (47.8%)	1.51 (0.96~2.37)	0.077^*^	28 (44.4%)	1.26 (0.74~2.14)	0.403
**No^#^**	311 (60.4%)	48 (52.2%)			35 (55.6%)		

Variables with p < 0.1 were subsequently enrolled into multivariate binary logistic regression analysis. ^*^Univariate regression analysis. ^#^The last category in the univariate regression analysis is the reference category. OR, odds ratio; BMI, body mass index; WBC, white blood cell; ANC, absolute neutrophil count; G-CSF, granulocyte colony-stimulating factor; BCS, breast-conserving surgery; SLNB, sentinel lymph node biopsy; ALND, axillary lymph node dissection; HR, hormone receptor; Her-2, human epidermal growth factor receptor 2; TNBC, triple-negative breast cancer; 4EC-4T, 4-period Epirubicin combined cyclophosphamide followed by 4-period Taxotere; 4TC, 4-period Docetaxel combined with cyclophosphamide; 6TC, 6-period Taxotere combined with cyclophosphamide; 6TCbH, 6-period Docetaxel combined with carboplatin and Trastuzumab. The bold values mean Univariate regression analysis p < 0.1.

### 3.2 The Variation Tendency of Peripheral Leukocyte and Absolute Neutrophil Counts

#### 3.2.1 The Variation Tendency of Peripheral White Blood Cell Counts and Absolute Neutrophil Counts in Different Regimens During Chemotherapy Cycles

The variation tendency of WBC counts and ANCs during the cycles of different regimens of adjuvant and neoadjuvant chemotherapies is presented in **Table 2**. Except in the 4-period Docetaxel and cyclophosphamide (4TC) group, the WBC counts and ANCs showed a decreasing tendency during the cycles. However, the overall mean of each group before each cycle of chemotherapy remained at normal levels. Therefore, prophylaxis with G-CSF can effectively maintain the levels of WBC counts and ANCs during chemotherapy and ensure that the treatment is carried out on time with a sufficient dose.

**Table 2:** The variation tendency of peripheral WBC counts and ANCs in different regimens during chemotherapeutic cycle.

Chemotherapy regimensChemotherapy cycle	4EC-4T		4TC		6TC		6TCbH	
	WBC (mean ± SD)	ANC (mean ± SD)	WBC (mean ± SD)	ANC (mean ± SD)	WBC (mean ± SD)	ANC (mean ± SD)	WBC (mean ± SD)	ANC (mean ± SD)
Cycle 1	6.47 ± 1.69	4.02 ± 1.38	6.25 ± 1.57	3.81 ± 1.36	6.81 ± 1.59	4.44 ± 1.41	6.48 ± 1.43	4.08 ± 1.25
Cycle 2	7.40 ± 2.76	5.01 ± 2.40	6.27 ± 1.85	4.11 ± 1.63	6.51 ± 1.57	4.41 ± 1.30	6.18 ± 2.33	3.93 ± 1.98
Cycle 3	6.96 ± 2.74	4.83 ± 2.51	6.54 ± 2.22	4.49 ± 1.94	6.55 ± 1.52	4.61 ± 1.39	5.73 ± 1.91	3.52 ± 1.55
Cycle 4	6.61 ± 3.05	4.58 ± 2.77	6.45 ± 2.09	4.47 ± 1.88	6.64 ± 1.95	4.79 ± 1.72	5.32 ± 1.87	3.22 ± 1.64
Cycle 5	6.55 ± 3.15	4.62 ± 2.89			6.27 ± 1.95	4.46 ± 1.70	5.87 ± 2.60	3.79 ± 2.43
Cycle 6	5.87 ± 2.56	4.16 ± 2.45			5.96 ± 2.14	4.32 ± 1.97	5.50 ± 1.72	3.52 ± 1.60
Cycle 7	5.76 ± 2.15	3.94 ± 1.96						
Cycle 8	5.79 ± 1.89	3.94 ± 1.74						

The data represent the white blood cell counts and the absolute neutrophil counts before each cycle of chemotherapy under the treatment of four different chemotherapy regiments. Data are expressed as mean ± SD. 4EC-4T, 4TC, 6TC, and 6TCbH represent different chemotherapy regiments. The levels of WBC counts and ANCs show a decreasing tendency with the number of chemotherapy cycle but remain stable within the normal range. WBC, white blood cell; ANCs, absolute neutrophil counts; 4EC-4T, 4-period Epirubicin combined cyclophosphamide followed by 4-period Taxotere; 4TC, 4-period Docetaxel combined with cyclophosphamide; 6TC, 6-period Docetaxel combined with cyclophosphamide; 6TCbH, 6-period Taxotere combined with carboplatin and Trastuzumab.

All patients prophylactically received G-CSF. Before the last cycle of chemotherapy, their overall mean WBC levels were significantly lower compared to baseline but remained stable within normal ranges (WBC, last baseline = −0.3864, t = −3.742, *p* < 0.001). The overall mean of ANC before the last cycle of chemotherapy did not significantly differ from the baseline (ANC, last baseline = 0.0625, t = 0.653, *p* = 0.514 > 0.05). Therefore, after prophylactic administration of G-CSF, neutrophil levels can be maintained at normal or even baseline levels during chemotherapy in ESBC patients, ensuring adequate dose and scheduled cycle of the treatment.

#### 3.2.2 The Variation Tendency of Peripheral White Blood Cell Counts and Absolute Neutrophil Counts in the 12-Month Follow-Up Period After Chemotherapy

**Figure 2:**
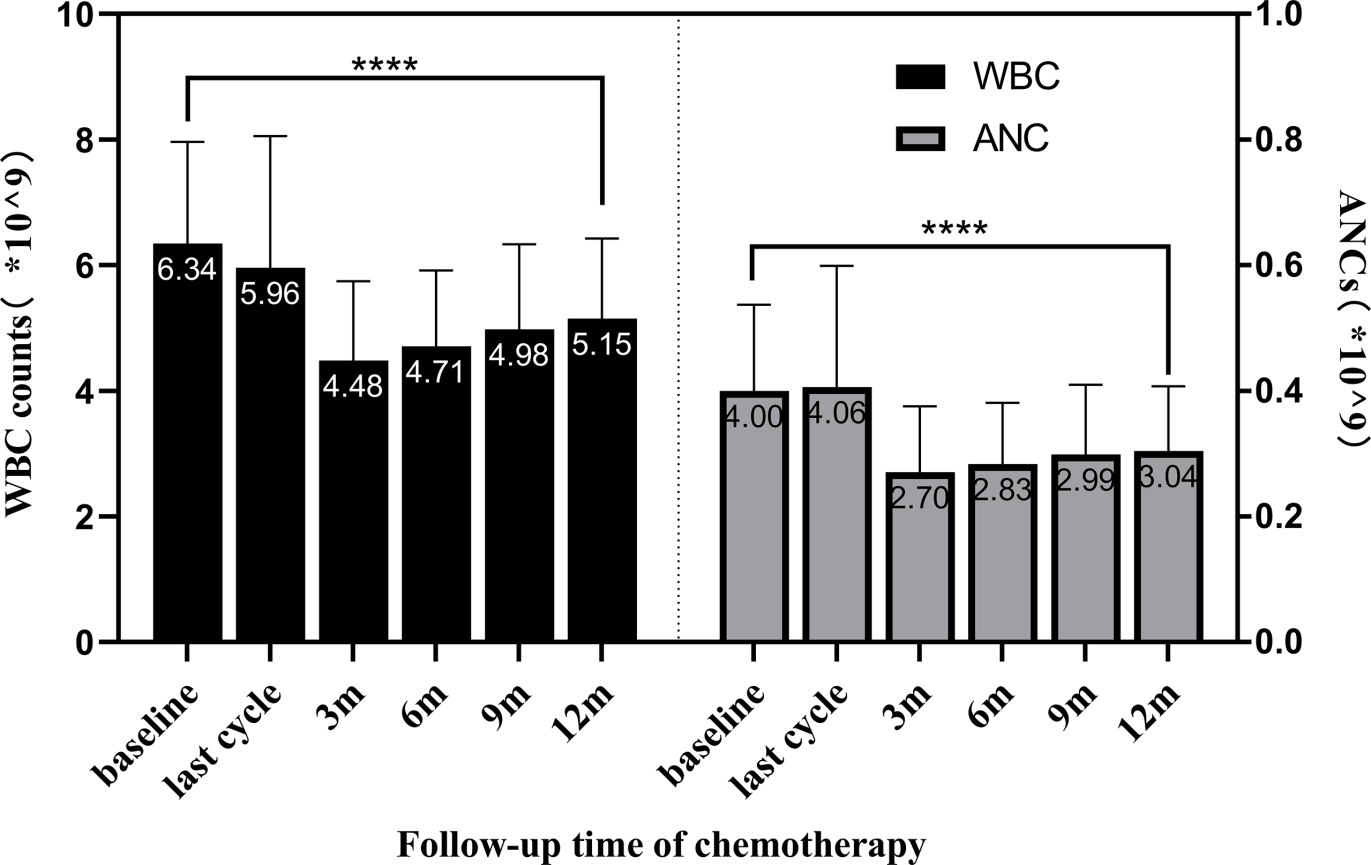
The variation tendency of WBC counts and ANCs in the 12-month follow-up after chemotherapy. Bars show the means ± SDs of WBC counts and ANCs at different time points. Peripheral WBC counts and ANCs gradually decreased to the lowest level after chemotherapy; however, they gradually recovered as the follow-up time increased. At 12 months after the end of the last chemotherapy, the overall mean of WBC counts was in the normal range but significantly lower than baseline (*p* < 0.0001 indicated by ****). WBC, white blood cell; ANCs, absolute neutrophil counts.

The means, SDs, and comparisons of WBC counts and ANCs levels between different time points are shown in **Table 3**. The variation tendency of peripheral WBC counts and ANCs during the 12-month follow-up period after the last chemotherapy is presented in **Figure 2**. After chemotherapy, peripheral WBC counts and ANCs gradually decreased to the lowest level. As the follow-up time increased, they gradually recovered. All patients received prophylaxis with G-CSF, but, at the 12th month after the end of the last chemotherapy, their overall mean levels of WBC counts and ANCs remained significantly lower than baseline (WBC, 12 months-baseline = −1.1911, t = −17.265, *p* < 0.0001; ANC, 12 months-baseline = −0.9514, t = −15.215, *p* < 0.0001).

**Table 3:** The changes of WBC counts and ANCs in follow-up period.

Follow-up period	WBC (mean ± SD)	ANC (mean ± SD)
**Prechemotherapy (baseline)**	6.34 ± 1.62	4.00 ± 1.38
**Prior to the last cycle of chemotherapy**	5.96 ± 2.10	4.06 ± 1.93
**3 months after chemotherapy**	4.48 ± 1.26^*#^	2.70 ± 1.05^*#^
**6 months after chemotherapy**	4.71 ± 1.20^*#^	2.83 ± 0.98^*#^
**9 months after chemotherapy**	4.98 ± 1.35^*#^	2.99 ± 1.11^*#^
**12 months after chemotherapy**	5.15 ± 1.27^*#^	3.04 ± 1.03^*#^

WBC, white blood cell; ANCs, absolute neutrophil counts.

^*^p < 0.05 compared with the baseline level (prechemotherapy level); a multi-pair sample non-parametric (Friedman) test.

^#^p < 0.05 compared with the level measured prior to the last cycle of chemotherapy; A multi-pair sample non-parametric (Friedman) test.

#### 3.2.3 The Variation Tendency of Peripheral White Blood Cell Counts and Absolute Neutrophil Counts During the 12-Month Follow-Up After Chemotherapy in Patients Prophylactically Receiving Different Granulocyte Colony-Stimulating Factor Types

According to the different G-CSF types used during chemotherapy, we divided the 515 ESBC patients into three different treatment groups: short-acting (used only rhG-CSF), long-acting (used only PEG-rhG-CSF), and mixed (used rhG-CSF or PEG-rhG-CSF in different cycles) groups.

**Figure 3:**
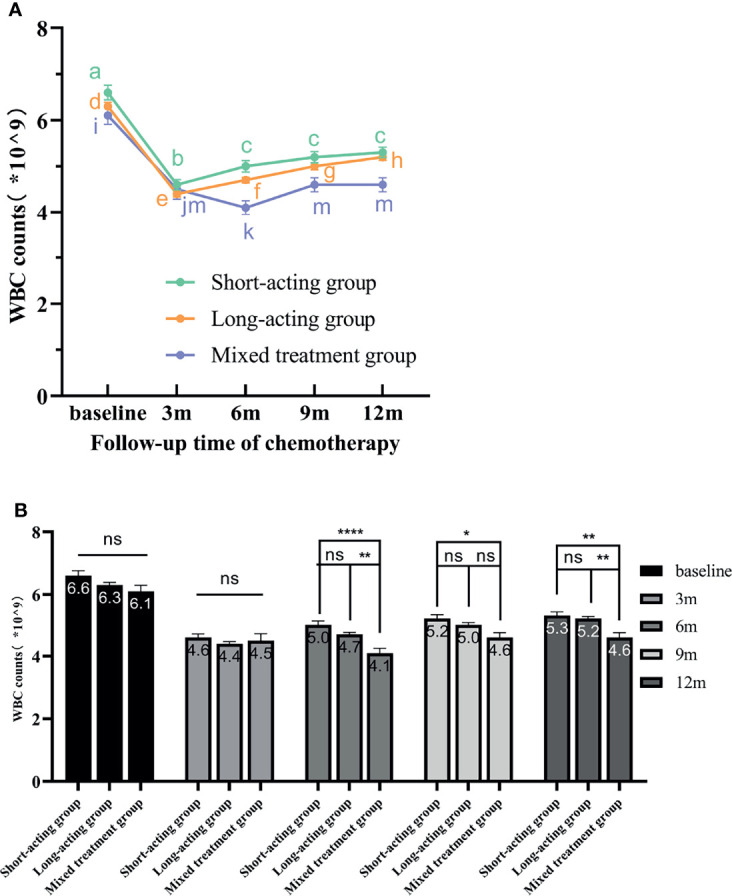
The variation tendency of WBC counts in different G-CSF treated groups. **(A)** The line chart exhibits the WBC count recovery trend from the three different G-CSF treatment groups at different time points. The different letters (a–m) on the line chart indicate significant intra-group differences (*p* < 0.05). **(B)** Bars represent the WBC counts means ± standard error of the mean (SEM) from the three treatment groups. “ns” indicates that there are no significant inter-group differences. * indicates significant inter-group differences at each time point (*p* < 0.05 indicated by *, *p* < 0.01 indicated by **, and *p* < 0.0001 indicated by ****). WBC, white blood cell; rhG-CSF, recombinant human granulocyte colony-stimulating factor; PEG-rhG-CSF, pegylated recombinant human granulocyte colony-stimulating factor; G-CSF, granulocyte colony-stimulating factor; SEM, standard error of the mean.

Based on the repeated-measures ANOVA results for WBC count, we found a significant inter-group effect for G-CSF treatment types. Different G-CSF treatments presented significant differences in the recovery of WBC counts during the follow-up period after chemotherapy (F = 6.206, *p* = 0.002). No significant difference was detected in the pairwise comparison regarding the recovery of WBC levels between long-acting and short-acting groups. However, these groups significantly differed from the mixed treatment group. Furthermore, both long-acting (*p* = 0.012) and short-acting (*p* = 0.000467) groups presented excellent increases in WBC counts compared to the mixed treatment group (**Figure 3A**).

Additionally, a significant intra-group effect was detected (**Figure 3A**); that is, within the same G-CSF treatment group, there were significant differences in WBC levels at different time points (F = 124.530, *p* < 0.00001). With the extension of follow-up time, the patient’s WBC level gradually recovered. The recovery of WBC counts in different G-CSF treatment groups is shown in **Figure 3**.

In the short-acting group, WBC levels increased gradually from 3 months after the end of chemotherapy as the follow-up time increased. Compared with baseline and 3 months after the end of chemotherapy, the WBC levels increased at 6, 9, and 12 months after chemotherapy and significantly differ. However, the increasing effect of WBC counts at 6, 9, and 12 months after the end of chemotherapy was not obvious, and there was no statistically significant difference. Therefore, these results indicated that, in the short-acting group, the WBC level recovered earlier, but the subsequent recovery rate was slow. In the long-acting group, from 3 months after the end of chemotherapy, WBC levels significantly and gradually increased along the follow-up time. The WBC levels also significantly differed between each follow-up time point. These results demonstrated that, in the long-acting group, WBC recovered earlier, faster, and better. Moreover, in the mixed treatment group, WBC recovered gradually over time but not in a linear manner. The WBC recovered from 6 months after the end of chemotherapy and stalled at 9 months. No significant difference in WBC levels was detected at 9 and 12 months after the end of chemotherapy. Hence, these results showed that the recovery effect of WBC was poor in the mixed treatment group.

The repeated-measures ANOVA also revealed a significant interaction between time points and different G-CSF treatment groups (F = 7.205, *p* = 0.000012). The WBC levels at baseline or 3 months after the end of chemotherapy did not significantly differ among G-CSF treatment groups. From 6 months after the end of chemotherapy, the long-acting and short-acting groups had better effects in promoting the recovery of WBC counts than the mixed treatment group along the follow-up time.

**Figure 4:**
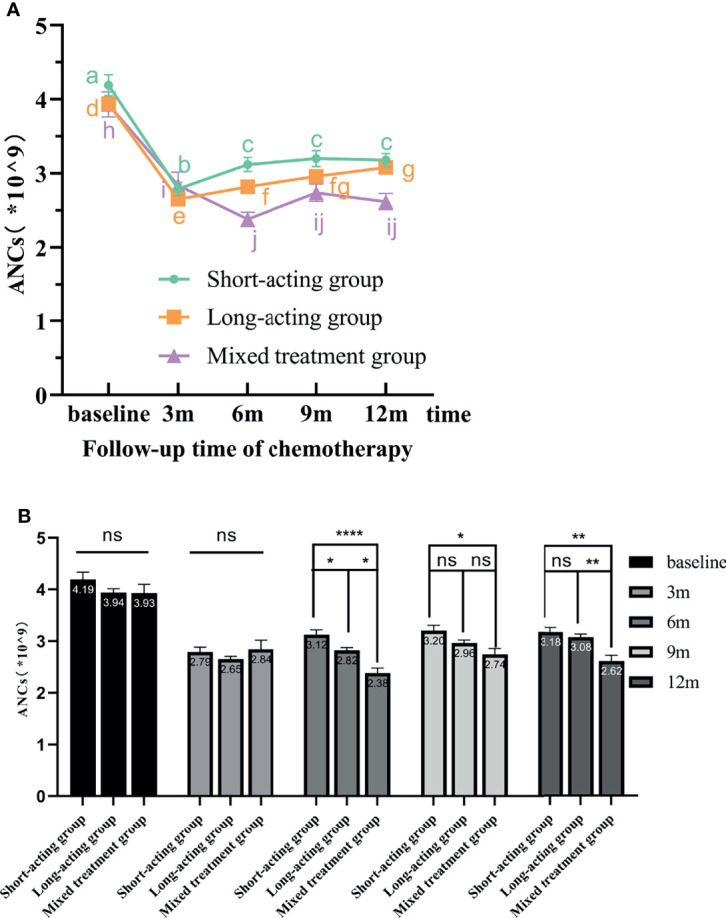
The variation tendency of ANCs in different G-CSF treated groups. **(A)** The line chart shows the recovery tendency of ANCs in the three different G-CSF treatment groups. The different letters (a–j) on the line chart indicate significant intra-group differences at various time points (*p* < 0.05). **(B)** Bars represent the ANCs means ± standard error of the mean (SEM) from the three G-CSF groups. “ns” indicates that there are no significant inter-group differences. * indicates significant inter-group differences at each time point (*p* < 0.05 indicated by *, *p* < 0.01 indicated by **, and *p* < 0.0001 indicated by ****). ANCs, absolute neutrophil counts; rhG-CSF, recombinant human granulocyte colony-stimulating factor; PEG-rhG-CSF, pegylated recombinant human granulocyte colony-stimulating factor; G-CSF, granulocyte colony-stimulating factor; SEM, standard error of the mean.

According to the repeated-measures ANOVA for ANCs, a significant inter-group effect was detected in the G-CSF treatment groups; in other words, different G-CSF treatment groups presented significant differences in the recovery of ANCs during the follow-up period after chemotherapy (F = 5.251, *p* = 0.006). No significant differences were detected in the pairwise comparison for the recovery of ANC levels between the long-acting and mixed treatment groups. However, these two groups were significantly different from the short-acting group. Moreover, the increases in ANC in the short-acting group were better than in the long-acting (*p* = 0.019) and mixed (*p* = 0.002) groups (**Figure 4A**).

The repeated-measures ANOVA also indicated a significant intra-group effect; that is, within the same G-CSF treatment group, significant differences in ANCs were detected at different time points (F = 77.256, *p* < 0.00001). Along the follow-up time, the patient’s ANC level gradually recovered. The recovery of ANCs for different G-CSF treatment groups is shown in **Figure 4**.

In the short-acting group, the ANCs increased gradually along the follow-up time from 3 months after the end of chemotherapy. Compared with baseline and 3 months after the end of chemotherapy, ANCs at 6, 9, and 12 months statistically differed and gradually increased. However, the ANC increase effects at 6, 9, and 12 months after the end of chemotherapy were not significant. Therefore, in the short-acting group, the ANC level recovered earlier and better, but the rate of subsequent recovery was slower. In the long-acting group, as the follow-up time increased, the ANC level gradually and significantly increased from 3 months after the end of chemotherapy. However, the increasing effect at 9 months after the end of chemotherapy was not as good as before. As a consequence, in the long-acting group, ANC levels recovered early, quickly, and continuously and had a good recovery effect. In the mixed treatment group, ANC recovered gradually over time but not in a linear manner. ANCs recovered from 6 months after the end of chemotherapy and stalled at 9 months. No significant differences were detected in the ANC level at 9 and 12 months after the end of chemotherapy. Therefore, these results indicated that the ANC recovery was slow and poor in the mixed treatment group.

The repeated-measures ANOVA for the ANCs also revealed a significant interaction between time points and different G-CSF treatment groups (F = 6.922, *p* = 0.000020). In other words, there were significant differences in the ANC level measured at different time points and G-CSF treatment groups. The ANC level at baseline or 3 months after the end of chemotherapy was not significantly different among G-CSF treatment groups. From 6 months after the end of chemotherapy, along with the follow-up time, the short-acting group had the best effect in promoting ANC recovery, followed by the long-acting group. The mixed treatment group had the worst effect (**Figure 4B**).

In summary, the repeated-measures ANOVA results indicated that regular prophylactic administration of the same G-CSF type during chemotherapy can effectively promote the recovery of WBC counts and ANCs after the treatment. Patients who had poor compliance and irregularly G-CSF use during the chemotherapy cycle were not conducive to the recovery of WBC and ANC after treatments.

### 3.3 Independent Influencing Factors of Long-term Leukopenia or Neutropenia after Chemotherapy

According to the data above, we found that WBC and ANC levels of some patients did not return to the baseline level even 12 months after chemotherapy or were even significantly lower than the normal threshold value, resulting in leukopenia or neutropenia. Although many studies explored CIL and CIN, they were mostly limited to the chemotherapy cycle of breast cancer patients. On the other hand, the recovery of WBC counts and ANCs after chemotherapy and the duration of leukopenia/neutropenia were rarely studied. Therefore, we explored the independent factors influencing WBC counts and ANCs after chemotherapy and the duration of leukopenia and neutropenia over 12 months.

We performed univariate regression analysis on 10 possible influencing factors, including age, BMI, different G-CSF types, baseline levels of WBC and ANC before treatment, surgical method, pathological type, molecular typing, lymph node metastasis, chemotherapy regimen, and Herceptin use. Variables with a *p* < 0.1 were included in the multivariate binary logistic regression model. The basic clinical characteristics of the 515 patients and the results of univariate analysis are detailed in **Table 1**.

Through the univariate regression analysis of leukopenia, five univariate factors had statistical significance (*p* < 0.1), including the baseline levels of WBC before treatment (*p* < 0.001), different G-CSF types (*p*_1_ = 0.006, *p*_2_ = 0.019), surgical method (*p* = 0.017), lymph node metastasis (*p*_1_ = 0.033, *p*_2_ = 0.054), and use of Herceptin (*p* = 0.077) (**Table 1**). These five univariate factors were included in the multivariate logistic regression analysis regarding the leukopenia duration 12 months after chemotherapy.

According to a multivariate logistic regression model, the independent factors influencing leukopenia in the 12th month after the end of the last chemotherapy are detailed in **Table 4**. These results indicated that the baseline level of WBC counts before treatment was a protective factor for leukopenia in the 12th month after the end of the last chemotherapy (*p* < 0.001). The baseline level of WBC counts was negatively correlated with the occurrence of leucopenia. The probability ratio of leukopenia was 0.468 between patients with a WBC counts one unit higher before treatment and patients one unit lower [*p* < 0.001, B = −0.760, odds ratio (OR) = 0.468, 95% CI: 0.379~0.577]. Moreover, different G-CSF types also affected the occurrence of leukopenia (*p* = 0.027). Among them, the probability of leukopenia in the short-acting group was lower than that in the mixed treatment group. The probability ratio of leukopenia between the short-acting group and the mixed treatment group was 0.380 (*p* = 0.027, B = −0.967, OR = 0.380, 95% CI: 0.162~0.895). The surgical method was also an independent influencing factor for leukopenia (*p* = 0.041). The probability of leukopenia in patients undergoing mastectomy and sentinel lymph node biopsy (SLNB) was lower than those undergoing mastectomy and axillary lymph node dissection (ALND). The probability ratio of leukopenia after chemotherapy in patients undergoing mastectomy and SLNB versus mastectomy and ALND was 0.470 (*p* = 0.041, B = −0.755, OR = 0.470, 95% CI: 0.228~0.968).

**Table 4:** Multivariate logistic regression model estimates of independent influencing factors for leukopenia in the 12th month after the end of the last chemotherapy.

Independent influencing factors	OR	95% CI	*p*-Value
**Baseline levels of WBC counts before treatment**	0.468	0.379~0.577	<0.001
**Different types of G-CSF**	0.380	0.162~0.895	0.027
**Surgical method**	0.470	0.228~0.968	0.041

OR, odds ratio; WBC, white blood cell; G-CSF, granulocyte colony-stimulating factor.

In the univariate regression analysis of neutropenia in the 12th month after the end of the last chemotherapy, the baseline levels of ANC before treatment (*p* < 0.001), different G-CSF types (*p*_1_ = 0.053, *p*_2_ = 0.049), and molecular typing (*p* = 0.022) were statistically significant factors (*p* < 0.1) (**Table 1**). These three univariate factors were used in the multivariate logistic regression analysis regarding neutropenia duration 12 months after chemotherapy.

The baseline level of ANC before treatment was a protective factor for neutropenia in the 12th month after the end of the last chemotherapy (*p* < 0.001) (detailed in **Table 5**). The baseline level of ANC was negatively correlated with neutropenia occurrence. The probability ratio of neutropenia was 0.431 between patients with ANCs one unit higher and patients one unit lower before treatment (*p* < 0.001, B = −0.841, OR = 0.431, 95% CI: 0.320~0.581). Moreover, different G-CSF types influenced the occurrence of neutropenia (*p* = 0.043). Among them, the probability of neutropenia in the long-acting group was lower than that in the mixed one. The probability ratio of neutropenia between the long-acting group and the mixed treatment group was 0.461 (*p* = 0.043, B = −0.773, OR = 0.461, 95% CI: 0.218~0.975). The molecular typing was also an independent factor influencing neutropenia (*p* = 0.025). Patients with hormone receptor (HR)-positive and human epidermal growth factor receptor 2 (Her-2) positive) typing were more likely to have neutropenia than triple-negative patients. The probability ratio of neutropenia between HR-positive (Her-2 positive) and triple-negative mesocytopenia was 3.147 (*p* = 0.025, B = 1.147, OR = 3.147, 95% CI: 1.157~8.560).

**Table 5:** Multivariate logistic regression model estimates of independent influencing factors for neutropenia in the 12th month after the end of the last chemotherapy.

Independent influencing factors	OR	95% CI	*p*-Value
**Baseline levels of ANCs before treatment**	0.431	0.320~0.581	<0.001
**Different types of G-CSF**	0.461	0.218~0.975	0.043
**Molecular typing**	3.147	1.157~8.560	0.025

OR, odds ratio; ANC, absolute neutrophil count; G-CSF, granulocyte colony-stimulating factor.

## 4 Discussion

Adjuvant and neoadjuvant chemotherapies are crucial for ESBC patients. Besides, CIL and CIN are significant issues in the safety management of chemotherapy (20). They are severe side effects during chemotherapy, which typically result in dose reduction and delay or interruption of the treatment cycle, thereby affecting its effectiveness in patients (21, 22). The effectiveness of chemotherapy is closely related to its RDI (23), and the occurrence of CIN or FN is a momentous event that limits the dose intensity of chemotherapy (24, 25). In the G-CSF guidelines, chemotherapy regimens containing platinum, anthracycline, or paclitaxel are high-risk factors for CIN. The administration of G-CSF for primary or secondary prophylactic treatments of CIN can ensure the safety and effectiveness of chemotherapy (26, 27).

In our hospital, the chemotherapy regimens used by ESBC patients generally contain epirubicin, platinum, or paclitaxel (high-risk factors for CIN). In the present study, ESBC patients received either prophylaxis with 3 or 6 mg of PEG-rhG-CSF or continuous 5–7 injections of rhG-CSF (100 μg) 48 h after the end of chemotherapy. The prophylactic use of G-CSF can effectively reduce the incidence of CIN and even FN to ensure the dose intensity and timeliness of chemotherapy (28). Lambertini et al. (15) revealed that PEG-rhG-CSF had better therapeutic effects and led to a shorter duration of CIN. Nevertheless, Li et al. (16) reported that, compared with rhG-CSF, PEG-rhG-CSF has no significant superiority for the security and effectiveness of CIN treatment and only reduces the pain of injections, thereby improving treatment compliance and the patients’ quality of life. The purpose of our study was to investigate whether PEG-rhG-CSF or rhG-CSF could prevent the incidences of CIL and CIN in ESBC patients during adjuvant or neoadjuvant therapies and whether it could effectively ensure the dose intensity cycle time of chemotherapy. We detected that the prophylactic employment of both long-acting and short-acting G-CSF could effectively ensure the relative stability of WBC counts and ANCs and kept these parameters in normal ranges in ESBC patients undergoing chemotherapy, guaranteeing the smooth progress of the chemotherapy cycle. However, since the chemotherapy cycle was carried out on time, except for the chemotherapy group (4TC), the mean values of WBC counts and ANCs in the other three regimens decreased. Moreover, the WBC counts before the last chemotherapy significantly differed from the baseline before treatment (*p* < 0.001).

Most of the current research has focused on the occurrence of CIN and FN during chemotherapy (29, 30), whereas few have studied the recovery tendency of WBC counts and ANCs and the duration of leukopenia and/or neutropenia after the end of the last chemotherapy. Neutrophils are vital components of the inflammatory response and innate immunity, comprehending a significant defense barrier against infections (31). Neutropenia is one of the most common and severe hematologic toxicity of cancer myelosuppressive chemotherapy, whose severity and duration not only limit the dose intensity of the treatment but also increase the risk of infections and even death (6). Therefore, it is imperative to explore the recovery of WBC counts and ANCs after chemotherapy and how to ensure the smooth progress of chemotherapy cycles and the effective recovery of these parameters after the treatment.

Here, all patients received G-CSF for prophylaxis during each cycle of chemotherapy. However, the employment of different G-CSF types affected the recovery of WBC counts and ANCs during the follow-up period after the end of the last chemotherapy. Through the line chart and repeated-measures ANOVA, we found that the WBC counts increased linearly after the end of the last chemotherapy in the long-acting and short-acting G-CSF groups, but the recovery effect was poor in the mixed G-CSF group (**Figure 3A**). Moreover, the results also suggested that the ANCs increased linearly after the end of the last chemotherapy, but the upward tendency and effect of the short-acting group were the best (**Figure 4A**), similar to the results of Mackey et al. Chemotherapeutic cytotoxicity can block cell division, affecting the rapid division of neutrophil progenitors in the BM, finally resulting in circulating blood neutropenia (32). The time required for neutrophil maturation within the BM is 6–7 days when the circulating neutrophils reach their lowest point (33). Mackey et al. (34) discovered that the timing of G-CSF used after chemotherapy was consistent with the initial decline in neutrophils counts (about 6–7 days after chemotherapy), which could better control the lowest point of neutropenia and neutrophil rebound. Therefore, delaying G-CSF treatment to 6–7 days after chemotherapy was recommended to improve circulatory ANCs more effectively. In our current study, patients in the short-acting group were injected with rhG-CSF once a day for 5-7 days at 24-48 hours after chemotherapy. Hence, the time of injection coincided with the dropping trend of neutrophil counts, when the effect of increasing ANCs might be better, consistent with Mackey et al. (34) We also demonstrated that patients with lower WBC counts and ANCs at baseline should receive G-CSF regularly. The regular use of PEG-rhG-CSF or rhG-CSF throughout the cycle can effectively improve the recovery of WBC counts and ANCs after the end of the last chemotherapy. However, due to poor compliance and/or economic reasons, some patients could not regularly use the same G-CSF type throughout the course of chemotherapy, which partly affected the recovery of WBCs counts and ANCs after treatment. Additionally, our results indicated that the WBC counts and ANCs of patients still did not return to the baseline level 12 months after the end of chemotherapy. On the other hand, the mean values of WBC counts and ANCs were within the normal reference value range (**Figure 2**). This also revealed that patients still had a strong recovery ability of BM hematopoietic capacity after chemotherapy, whether they prophylactically used PEG-rhG-CSF or rhG-CSF.

Furthermore, we found that, compared with the last chemotherapy, along the follow-up time after the treatment, WBC counts and ANCs gradually recovered but did not reach baseline levels (*p* < 0.0001). More seriously, some patients still had leukopenia or neutropenia after 12 months of the end of the last chemotherapy. In our current study, 92 (17.9%) and 63 (12.2%) patients still suffered from leukopenia and neutropenia, respectively, in the 12th month after finishing the last chemotherapy cycle. Therefore, it is necessary to assess the factors influencing the recovery of WBC counts and ANCs in ESBC patients after chemotherapy. In G-CSF guidelines, Smith et al. (35) suggested that medical history, disease characteristics, chemotherapy regimen, and age are risk factors for leukopenia and neutropenia during chemotherapy. Moreover, Lyman et al. (19) reported that poor body condition, chemotherapy history, radiation therapy history, and neutropenia before chemotherapy are risk factors for neutropenia and even FN during chemotherapy. This indicated that the patient’s individual state is also an important factor affecting the recovery of WBC counts and ANCs. Therefore, we also included individual-related clinical characteristics that might affect leukocytes and neutrophils in univariate regression analysis of leukopenia and neutropenia 12 months after the end of the last chemotherapy. Five and three influencing factors were respectively selected for multivariate logistic regression analysis of leukopenia and neutropenia. We found three independent factors (WBC counts at baseline, G-CSF types, and surgical method) that influenced the duration of leukopenia and three independent factors (ANC at baseline, G-CSF types, and molecular typing) that influenced the duration of neutropenia after 12 months.

The WBC count and ANC at baseline reflect the patients’ BM hematopoietic capacity. Thus, patients with lower baseline levels of these parameters are more likely to develop leukopenia and neutropenia following myelosuppressive chemotherapy (36, 37). This is consistent with our findings that the baseline levels of WBC count and ANCs are protective factors for leukopenia and neutropenia. Moreover, patients in the mastectomy and ALND groups were more likely to develop leukopenia than the mastectomy and SLNB groups. This might be related to the fact that patients undergoing mastectomy and SLNB generally do not require postoperative adjuvant radiotherapy. However, patients undergoing ALND have more lymph node metastasis, and radiation therapy is required after adjuvant chemotherapy (38), which affects the recovery of WBCs and leads to a longer duration of leukopenia (39). We also detected that molecular typing affects the duration of neutropenia after chemotherapy, possibly because it determines, to a certain extent, the treatment for breast cancer. Early-stage triple-negative breast cancer (TNBC) patients rarely undergo further treatment after adjuvant chemotherapy, but ESBC patients with Her-2 and HR-positive still need endocrine (40) and targeted (41) therapy after chemotherapy, which also affects the recovery of ANCs and contribute to common side effects, including neutropenia (42, 43). In addition, the majority of chemotherapy regimens for breast cancer patients with positive HR and HER-2 contained platinum, such as 6-period Docetaxel combined with carboplatin and Trastuzumab (6TCbH) regimen in our research. Numbness of hands and feet caused by sensory nerve injury is a common side effect of chemotherapy in these patients (44). A study (45) has shown that the use of multi-cycle platinum chemotherapy not only can lead to sensory nerve injury but also may cause BM neuropathy. BM nerve injury results in chronic injury of BM stromal cells (BMSC), which weakens the hematopoietic production and reserve function of BM and even affects the mobilization of hematopoietic stem cells induced by G-CSF, thus impairing the recovery of neutrophils after chemotherapy.

Our results indicated that the risk of long-term leukopenia after chemotherapy in the short-acting group and the risk of long-term neutropenia after chemotherapy in the long-acting group were lower as compared to the mixed treatment group. However, no significant difference was detected for the incidence of leukopenia and neutropenia 12 months after chemotherapy between the long-acting and the short-acting groups. PEG-rhG-CSF and rhG-CSF have the same mechanism of action: G-CSF binds to G-CSF receptors expressed on hematopoietic cells to stimulate the proliferation of hematopoietic progenitor cells and accelerate the transformation of immature metamyelocytes into mature neutrophils, thereby increasing functional neutrophils in circulating peripheral blood (46, 47). Compared with rhG-CSF, PEG-rhG-CSF can decrease plasma clearance and prolong half-life (15). Huang et al. (48) and Park et al. (49) showed that PEG-rhG-CSF or rhG-CSF have similar efficacy to ameliorate neutropenia. Previous studies focused mainly on the duration of leukopenia and neutropenia during chemotherapy, while we focused on the follow-up period after chemotherapy. Our results manifested a little difference between the efficacy of PEG-rhG-CSF and rhG-CSF if dosed based on the recommended guidelines. This also suggested that during the chemotherapy cycle, continuous use of the same G-CSF type can better ensure the recovery of WBC counts and ANCs after chemotherapy and reduce the duration of leukopenia and neutropenia.

The above repeated-measures ANOVA and multivariate regression analysis indicated that differences in the long-term effects of the short-acting and the long-acting groups on WBC counts and ANCs were small during the follow-up after the end of the last chemotherapy, but there is no denying that the treatment outcome of the mixed treatment group was the worst. The mixed treatment group had the worst long-term recovery of WBC and ANC levels and had higher incidences of leukopenia and neutropenia after the end of the last chemotherapy. Therefore, we propose the following probable mechanisms for the cause of poor treatment effects of the mixed treatment group. For one thing, the main reasons for changing G-CSF treatment regimens in the mixed treatment group patients during the chemotherapy cycle were poor patient compliance and limited economic conditions. Patients with poor compliance may not strictly follow the doctor’s advice after being replaced with short-acting rhG-CSF, and patients may voluntarily reduce or delay the injection due to the increased number of rhG-CSF injections. Standard administration of G-CSF can promote the recovery of the hematopoietic function of BM and the protection of chemotherapy (50). However, the irregular use of G-CSF in the mixed treatment group combined with the damage of hematopoietic precursor cells and BM stroma caused by the toxicity of chemotherapy drugs may result in impaired secretion and dysfunction of G-CSF, thus bringing about the insufficient mobilization of hematopoietic stem cell and abnormal expression of adhesion molecules on relevant cells such as progenitor cells in the BM microenvironment (51, 52). Then, this can lead to the disorder of the BM microenvironment to some extent and give rise to the poor recovery effect of granulocytes. For another thing, patients in the mixed treatment group were generally switched to the cheaper rhG-CSF during the treatment cycle because they could not afford the cost of PEG-rhG-CSF due to limited economic conditions. Economic conditions often affect the nutritional status of patients after treatment. Hastreiter et al. (53) found that protein malnutrition can damage the regeneration and hematopoiesis of the BM, weaken patients’ response to the stimulation of G-CSF, which can cause the failure of the production of neutrophils, and may even affect the expression of the G-CSF receptors, thereby weakening the therapeutic outcome of G-CSF. Future research could add data on patients’ nutritional status after treatment, such as serum albumin concentration. Thirdly, the response of neutropenia to G-CSF is highly variable, such as irregular or late use of G-CSF in each chemotherapy cycle may contribute to destructive resonance in neutrophil dynamics, even permanent oscillation, thus leading to long-term neutropenia (54). Accordingly, future studies should focus on monitoring improvements during the follow-up period after chemotherapy, increasing the sample size and date feature and exploring the therapeutic opportunity and specific action mechanism of drugs.

## 5 Conclusion

Overall, the regularly prophylactic use of PEG-rhG-CSF or rhG-CSF can effectively stabilize WBC counts and ANCs during chemotherapy and ensure treatment’s RDI and tolerance. The alternating use of different G-CSF types during the cycle affected the recovery of WBC counts and ANCs after the end of the last chemotherapy. Long-term leukopenia and neutropenia after chemotherapy was associated with WBC count and ANC at baseline, G-CSF types, surgical method, and molecular typing. The application and management of G-CSF in ESBC patients after chemotherapy still need further research.

## Data Availability Statement

The original contributions presented in the study are included in the article/supplementary materials, further inquiries can be directed to the corresponding author.

## Ethics Statement

This study was reviewed and approved by the Institutional Review Board of the Second Affiliated Hospital of Zhejiang University School of Medicine. The ethics committee denoted that since this study was a retrospective cohort study, which did not involve human experiments, the informed consent of individual patients was not required.

## Author Contribution

WT and YD conceptualized the research project. YW drafted the manuscript. YW, YZ, and YY participated in the data collection. WT, YW, and YZ reviewed and modified the manuscript. YD supervised the research and led the discussion. All authors approved the final version of the manuscript.

## Conflict of Interest

The authors declare that the research was conducted in the absence of any commercial or financial relationships that could be construed as a potential conflict of interest.

## Publisher’s Note

All claims expressed in this article are solely those of the authors and do not necessarily represent those of their affiliated organizations, or those of the publisher, the editors and the reviewers. Any product that may be evaluated in this article, or claim that may be made by its manufacturer, is not guaranteed or endorsed by the publisher.

## Abbreviations

CIN, chemotherapy-induced neutropenia; FN, febrile neutropenia; rhG-CSF, recombinant human granulocyte colony-stimulating factor; PEG-rhG-CSF, pegylated recombinant human granulocyte colony-stimulating factor; ESBC, early-stage breast cancer; G-CSF, granulocyte colony-stimulating factor; WBC, white blood cell; ANCs, absolute neutrophil counts; OS, overall survival; DFS, disease-free survival; RDI, relative dose intensity; CIL, chemotherapy-induced leukopenia; BMI, body mass index; LSD, least significant difference; OR, odds ratio; SLNB, sentinel lymph node biopsy; ALND, axillary lymph node dissection; HR, hormone receptor; Her-2, human epidermal growth factor receptor 2; 4TC, 4-period Docetaxel and cyclophosphamide; TNBC, triple-negative breast cancer; 6TCbH, 6-period Docetaxel combined with carboplatin and Trastuzumab; BM, bone marrow; BMSC, bone marrow stromal cells.
